# Hemodynamic Responses during Enduro-Motorcycling Performance

**DOI:** 10.3389/fphys.2017.01062

**Published:** 2017-12-14

**Authors:** Irene Sanna, Virginia Pinna, Raffaele Milia, Silvana Roberto, Sergio Olla, Gabriele Mulliri, Antonio Crisafulli

**Affiliations:** Department of Medical Science and Public Health, Sports Physiology Laboratory, University of Cagliari, Cagliari, Italy

**Keywords:** heart rate, stroke volume, blood pressure, blood lactate, exercise pressor reflex

## Abstract

Much of the information available in the literature on physiological responses during Enduro motorcycling is related to heart rate (HR) and blood lactate (BLa). The aim of this work was to investigate the hemodynamic changes that occur during a 10-min session of Enduro motorcycling. Fifteen skilled riders were enrolled on the study and all participants underwent an Enduro-motorcycling session on a standard track. Hemodynamics were assessed using a miniaturized impedance cardiograph. Results show that HR significantly increased from 96.5 ± 12.8 bpm at rest to 153.1 ± 17.7 bpm during riding, while stroke volume (SV) increased from 53.5 ± 14.1 to 72.2 ± 22.1 ml and cardiac output (CO) from 5.0 ± 1.1 to 10.9 ± 3.0 L·min^−1^. Moreover, ventricular emptying rate (VER) increased from 192.9 ± 43.0 to 324.1 ± 83.6 ml·s^1^ and ventricular filling rate (VFR) from 141.1 ± 160.5 to 849 ± 309 ml·s^−1^. Taken together, these data suggest that Enduro motorcycling induces substantial cardiovascular activation, not only in terms of chronotropism but also in terms of cardiac performance and pre-load, thereby increasing SV and CO. Finally, it is likely that sympathetic-mediated venous constriction occurred. This in turn improved VFR and recruited the Frank-Starling mechanism and inotropic reserve. It was concluded that Enduro motorcycling is a challenging activity for the cardiovascular apparatus.

## Introduction

Very little information on the physiological responses exists. Almost all the information available is related to heart rate (HR) adjustment and blood lactate (BLa) accumulation (Gobbi et al., 2005; D'Artibale et al., 2007; Konttinen et al., 2007).

One category of motorcycling is Enduro, where races are held on country roads, mule tracks, and public roads under ordinary traffic rules (Gobbi et al., 2005). Competitions last from 6 to 8 h and riders do not know the route and may face unexpected obstacles. Distances between checkpoints must be covered in established times (i.e., with a fixed average speed), from the cardiovascular point of view, Enduro causes acute and unpredictable variations in HR and possibly to other hemodynamic parameters (such as cardiac pre-load, inotropism and after-load) yet to be described. Moreover, the long duration of Enduro races, together with the clothing and equipment, can further stress athletes' cardiovascular systems because of heat and dehydration. The limited data available report that HR can reach values close to 190 bpm (Gobbi et al., 2005), thereby suggesting an increase in sympathetic tone which leads to cardiovascular activation. Moreover, Enduro requires the recruitment of the aerobic metabolism, which can be maintained at a level above that of the anaerobic threshold (AT) (Gobbi et al., 2005).

It is well-known that during exercise, cardiovascular adjustments are caused by the activation of the central command and by mechanical and metabolic muscle activity, which evoke the exercise pressor reflex (Nóbrega et al., 2014). These cardiovascular reflexes are supposed to be activated during Enduro since this activity causes motor cortex activation and mechanical and metabolic engagement at the muscle level (Gobbi et al., 2005). Considering these features, it is reasonable to hypothesize that the cardiovascular apparatus is subjected to stress during Enduro performances. Furthermore, the activation of both central command and exercise pressor reflex recruits myocardial inotropism (Nóbrega et al., 2014) which increases stroke volume (SV). The combination of increments in SV and HR induces elevation in cardiac output (CO). However, to the best of our knowledge, nobody to date has measured CO during motorcycle riding.

Furthermore, it is well known that changes in HR and SV may oppose each other (Higginbotham et al., 1986; Crisafulli et al., 2011), hence their combined effect should be investigated. Although SV is not easily measured, this parameter has been successfully assessed recently by means of a portable impedance cardiograph, which is able to gather hemodynamic data in extreme environments, such as during underwater diving (Tocco et al., 2013; Marongiu et al., 2015).

This investigation was devised to study acute hemodynamic adjustments induced by Enduro riding by using trans-thoracic impedance. In detail, we were interested in obtaining a complete hemodynamic picture by measuring HR, SV, and CO during a real Enduro session of short duration. The hypothesis was that isometric strains occurring during Enduro impaired venous return, thereby reducing cardiac pre-load, and increased after-load, which opposed cardiac emptying. This phenomenon would result in the impossibility to increase SV despite the Enduro-induced activation in central command and exercise pressor reflex activation. These two reflexes, together with HR increments, are supposed to recruit cardiac performance and pre-load (Crisafulli et al., 2009; Roberto et al., 2012; Marongiu et al., 2013; Milia et al., 2014; Nóbrega et al., 2014). Thus, if static effort prevailed over dynamic effort in Enduro, a reduction of, or a stable SV value throughout the race would be expected.

## Methods

### Subjects

Fifteen male Enduro riders were enrolled. All riders had regularly participated in competitions over the previous 5 years. Mean ± standard deviation (SD) of age, height, and body mass were 32.33 ± 8.76 years, 175.47 ± 6.39 cm, and 76.80 ± 9.18 kg respectively. All subjects were skilled athletes who trained for 8–10 h a week and had been involved in regular training programs for at least 5 years. The study was conducted in accordance with the Declaration of Helsinki and was approved by the University Hospital of Cagliari ethics board. Written informed consent was obtained from all participants.

### Experimental design

#### Preliminary test

All subjects underwent a general medical examination followed by an incremental exercise test on an electro-magnetically-braked cycle ergometer with ECG monitoring (CUSTO Med, Ottobrunn, Germany) to exclude any cardiovascular problems. Maximum workload values achieved by subjects during the incremental test was 235.20 ± 14.80 w. These data indicate levels of aerobic fitness similar to those previously reported by our laboratory in soccer players performing the same test (Crisafulli et al., 2004).

#### Enduro session

**Figure 1:**
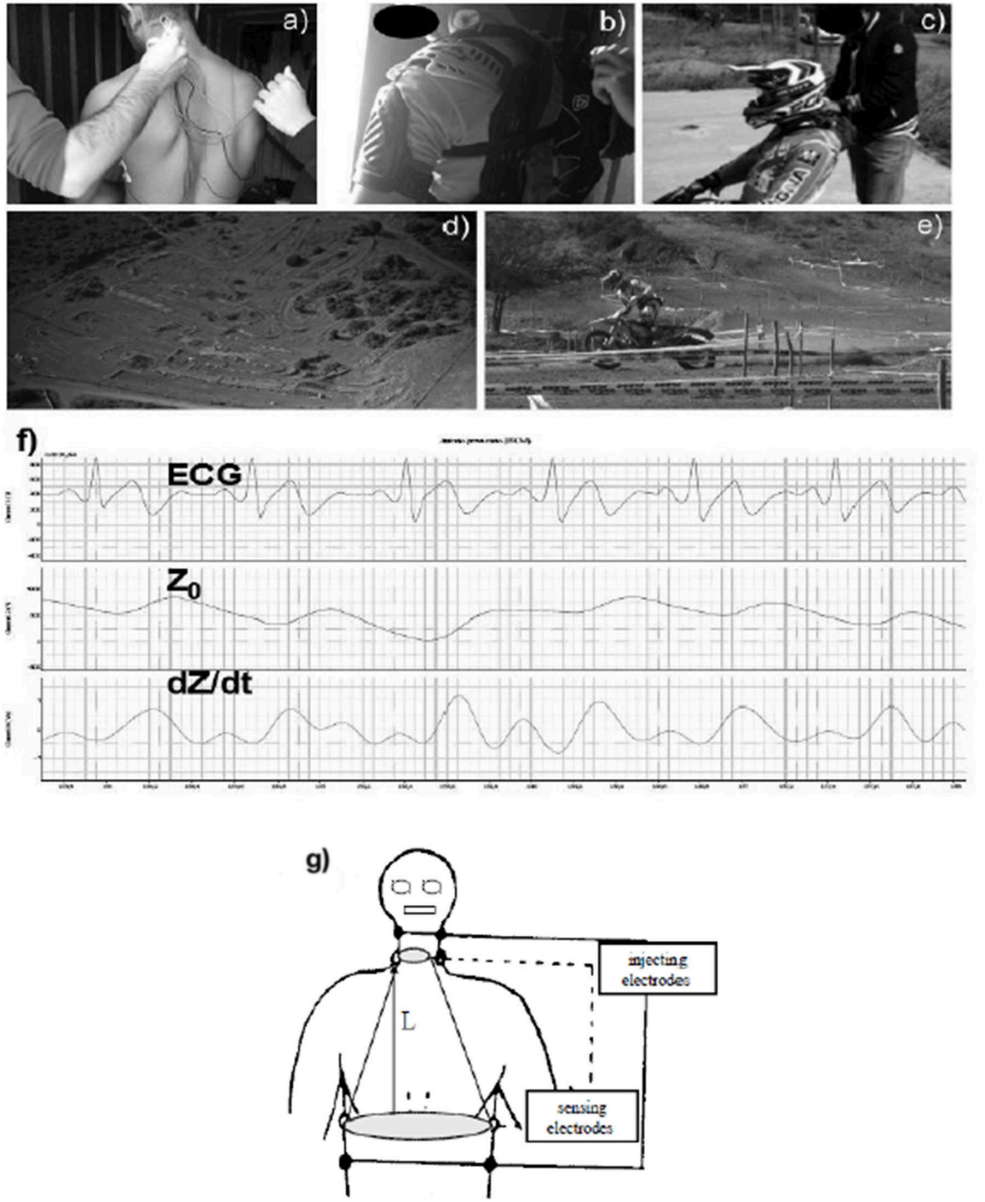
An example of a subject wearing the New Core device. **(a)** Shows the application of pairs of neck and thoracic electrodes. **(b,c)** Show the subject after wearing all the Enduro equipment. **(d)** Is an aerial picture of the Enduro track. **(e)** Shows the subjects while riding. Finally, **(f)** Is an example of analog electrocardiogram traces (ECG), thoracic impedance (Z0), and its first derivative (dZ/dt) recorded during the riding session. **(g)** Is a schematic representation of electrodes placement.

On a different day from the medical examination, all subjects underwent a short Enduro motorcycle session on a standard track, which was used regularly for training purposes. It was 986 meters long on natural terrain containing gravel, sand, and mud and had 5 artificial sandy bumps. Sessions took place between September and December. Rainy days were avoided. Air temperature was between 14 and 23°C (16.6 ± 2.8°C). Wind speed was always below 25 km/h (14.2 ± 3.6 km/h), as reported by the local weather bulletin. The session was not a real race. Instead, it was a situation similar to a training session, without the psychological stress imposed by a real race. The riders performed the test alone without rivals. This in order to avoid the bias due to competition-induced sympathetic activation. Participants were allowed to perform a 15-min warm-up prior to the session consisting in riding along the track at their preferred speed. After the warm-up and before the start of the Enduro session, a miniaturized impedance cardiograph with eight spot electrodes (New Core, 2C Technologies Inc., Cagliari, Italy) was connected to the subject. Dual lower-thoracic, voltage-sensing electrodes were placed perpendicularly to the longitudinal plane of the sternum, laterally to the xiphoid process in the mid-axillary line. Two cervical, voltage-sensing electrodes were placed as closely as possible to the clavicles at the lateral aspect of the base of the neck. The current-injecting electrodes (2.5 mA, 65 KHz) were placed 5 cm above the cervical-sensing electrodes and below the thoracic-sensing electrodes. Figure 1a shows a subject wearing the New Core device before starting the Enduro session. Figure 1g of the same figure shows the points of electrode placement. The New Core was placed in a bag that was secured to the back of the subject with a harness. Then, he sat on a chair for 5 min in order to assess data at rest. The Enduro session commenced after this rest period. In detail, the subject was asked to perform a total of 10 min of riding at the maximum speed possible, i.e., to cover the longest distance possible within a fixed time. When the Enduro session was completed, the athlete rested again on a chair for a further 5 min in order to recover. Panels b and c of Figure 1 show one subject wearing all the Enduro equipment. Panels d and e of the same figure show an aerial picture of the Enduro track and one subjects while riding.

The 10-min period of motorcycle trial was chosen to avoid cardiovascular perturbations due to the potential occurrence of cardiac drift (Crisafulli et al., 2006a), thermal stress, and dehydration (Cheuvront et al., 2010; González-Alonso, 2012). Actually, our study focused mainly on acute hemodynamic changes rather than on the cardiovascular consequences due to heat stress and dehydration.

Throughout rest, exercise, and recovery, hemodynamics were assessed by the impedance method, which is usually employed in hemodynamic measurement during resting, exercising, and recovering subjects (Charloux et al., 2000; Richard et al., 2001; Crisafulli et al., 2004, 2006b, 2007). The impedance method provides reliable, non-invasive data on thoracic fluid index (TFI), left ventricular ejection time (VET), SV, HR, and CO. Briefly, this technique assumes that, when an electrical current circulates through the thorax, the pulsate aortic blood flow causes a proportional fluctuation in the electrical conductivity. This in turn induces changes in thoracic electrical impedance during systole, which are representative of SV (Bernstein, 1986; Warburton et al., 1999). The New Core device recorded impedance and ECG traces throughout the experimental session on a secure digital memory card. The recorded impedance and ECG traces were then analyzed offline employing a digital chart recorder (ADInstruments, PowerLab 8sp, Castle Hill, Australia), and hemodynamic parameters were calculated. Figure 1f shows typical New Core-derived traces analyzed offline after recording. This data processing method has been employed several times recently and has been described in detail in previous papers (Crisafulli et al., 2000, 2003a, 2007, 2008; Tocco et al., 2013; Marongiu et al., 2015). In short, New Core-derived analog traces of ECG, thorax impedance (Z_0_), and its first derivative (dZ/dt) were stored and then analyzed offline to exclude signals affected by movement and respiratory artifacts. The precise procedure by which traces have been analyzed is detailed shown in our previous papers (Crisafulli et al., 2000, 2003a). Measurements were performed only in traces with at least 20% of the total beats are artifacts-free in 1 min of recording, i.e., at least 12 s of artifacts-free traces must have been present in 1 min recording to perform analysis.

This procedure, although time consuming, allows for fake signals from SV calculation to be excluded. The New Core device had been previously validated against a standard impedance cardiograph (NCCOM3-R7, BoMed, CA Inc.) and was found to be reliable (Tocco et al., 2013).

The SV was calculated by applying the following Sramek-Bernstein equation (Bernstein, 1986),

(1) $$\begin{array}{r} \text{SV}=\left(\text{VEPT}•{\text{Z}}_{0}^{-1}\right)•\text{dZ}/\text{d}{\text{t}}_{\text{max}}•\text{VET} \end{array}$$

In detail, VEPT was the volume of electrical participating tissue and was derived using a nomogram from subjects' sex, height, and weight. Z_0_ was the thorax impedance measured at the end of cardiac diastole (Crisafulli et al., 2003a); dZ/dt_max_ is the maximal Z_0_ first derivative value during cardiac systole. VET was the left ventricular ejection time, calculated as the interval between the beginning and the minimum of the deflection in dZ/dt trace during systole. HR was calculated as the reciprocal of the electrocardiogram R-R interval, and CO was obtained by multiplying SV •HR. The mean systolic ejection rate (VER), obtained by calculating the SV/VET ratio, was also assessed. This parameter was considered an index of myocardial performance (Tanaka et al., 1986; Concu and Marcello, 1993; Gledhill et al., 1994). Moreover, diastolic time (DT) was measured by subtracting the sum of the pre-ejection period (PEP, which was assessed as the time interval between the onset of the electrocardiogram Q wave and the beginning of the widest deflection occurring in the dZ/dt trace) and VET from the total cardiac cycle period. The ventricular filling rate (VFR), a measure of the mean rate of diastolic blood flux, was calculated by dividing SV by DT (Gledhill et al., 1994; Crisafulli et al., 2000, 2003a,b; Marongiu et al., 2013; Milia et al., 2014).

Arterial blood pressure was measured at rest and during recovery (at the third min) in a seated position by utilizing a standard manual sphygmomanometer. Systemic vascular resistance (SVR) was obtained by dividing mean blood pressure (MBP, calculated as diastolic blood pressure +1/3systolic blood pressure-diastolic blood pressure) by CO.

Blood samples were obtained with a finger prick. The skin where the blood was drawn was cleaned, sweat was dried using a cotton gauze, and finally disinfected. BLa concentration was measured at rest and within 1 min of the end of the Enduro session using a portable lactate analyser (Lactate Pro, Arkray Inc., Kyoto, Japan).

#### Statistical analysis

Data are reported as mean ± SD and were averaged over 1 min during rest and recovery. Variables at rest were considered those of the last min of rest preceding the motorcycle session, whilst variables at recovery were considered those of the third min of recovery. Data during the Enduro session were averaged over 1 min. The mean values reached during the last 3 min of the session (i.e., when a steady state should have been reached) were used for comparison with the other protocol sessions. Descriptive statistics were performed on each variable to confirm the assumptions of normality by means of the Kolmogorov-Smirnov test. The alpha level was set at *P* < 0.05. Comparisons between periods in hemodynamic parameters were carried out using one-way repeated measures analysis of variance (ANOVA), followed by the Neuman-Keuls *post-hoc* when appropriate. Differences between rest and recovery in BLa, MBP, and SVR were evaluated by means of the *t* test for paired data. Significance was set at a *p*-value of <0.05. Statistics were calculated employing commercially available software (Graph-Pad Prism, version 4.00, 2003).

## Results

**Figure 2:**
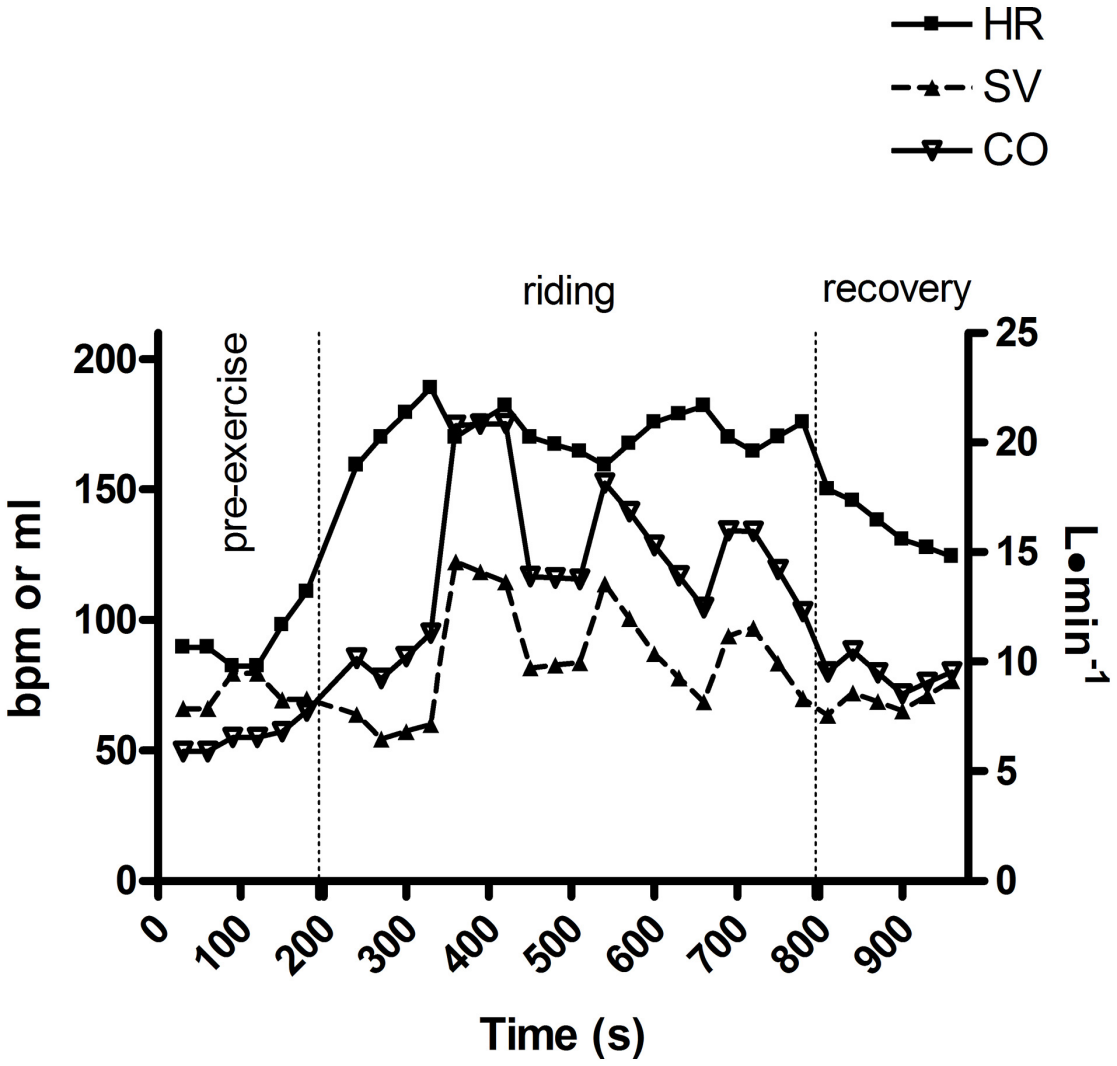
An example of heart rate (HR), stroke volume (SV), and cardiac output time course throughout an Enduro session in one subject.

All Subjects Completed the Study Protocol. Figure 2 shows an example of the time course of HR, SV, and CO in one subject throughout the test. This picture shows that cardiovascular response was intermittently activated. Results from this observation look mainly at the SV time course, rather than at HR. In reality, whilst the HR level increased and almost stabilized, SV showed several peaks throughout the session.

**Figure 3:**
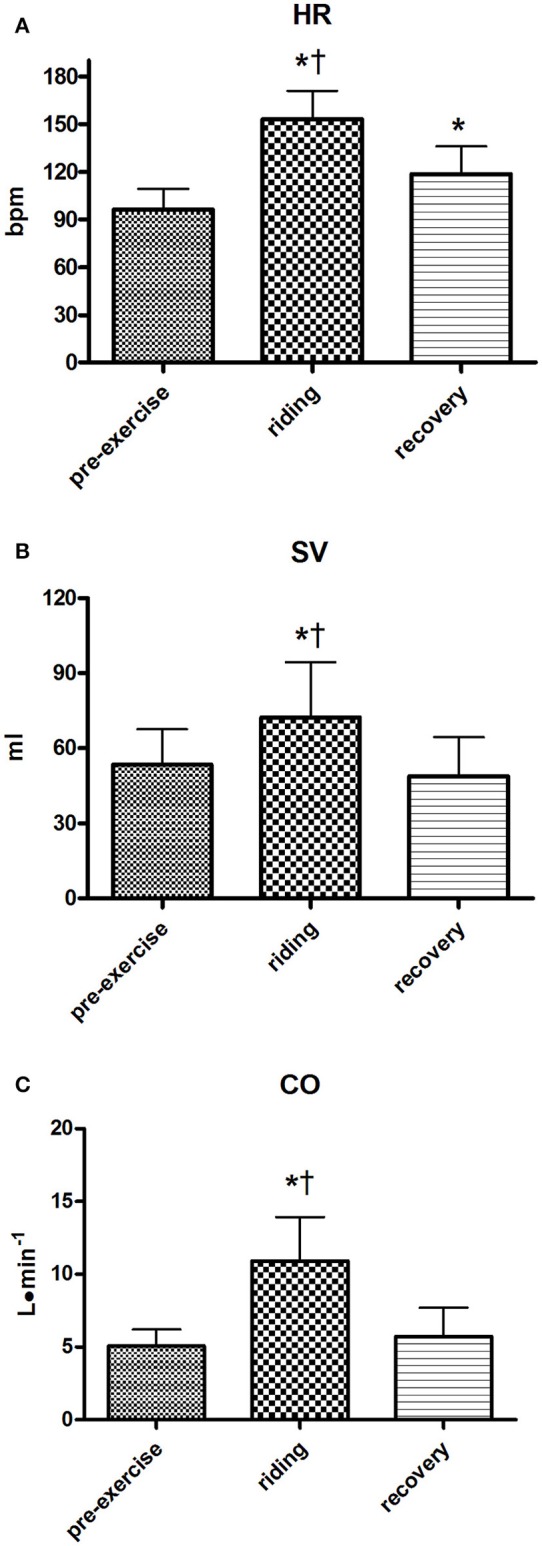
Group heart rate values (HR, **A**), stroke volume (SV, **B**), and cardiac output (CO, **C**) during the various periods of the Enduro session. Variables at rest were considered those of the last minute of rest preceding the motorcycle session, variables during riding were the average of the 10 min Enduro session, while variables at recovery were considered those of the third minute of recovery. Values are mean ± SD (*n* = 15). ^*^p < 0.05 vs. pre-exercise; ^†^*p* < 0.05 vs. recovery.

**Figure 4:**
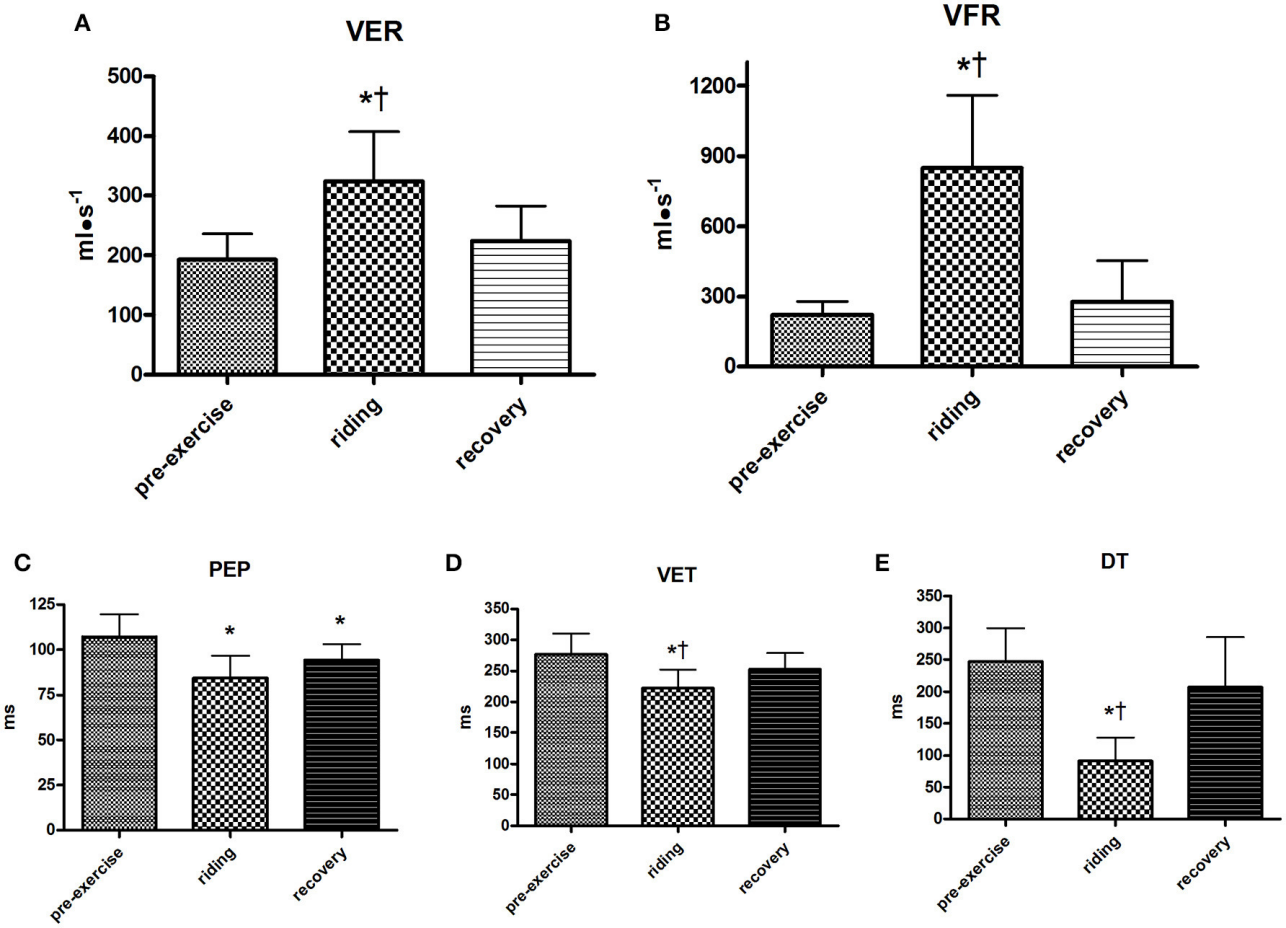
Group ventricular emptying rate (VER, **A**), ventricular filling rate (VFR, **B**), pre-ejection period (PEP, **C**), ventricular ejection time (VET, **D**), and diastolic time (DT, **E**) during the various periods of the Enduro session. Values are mean ± SD (*n* = 15). ^*^p < 0.05 vs. pre-exercise; ^†^*p* < 0.05 vs. recovery.

**Figure 5:**
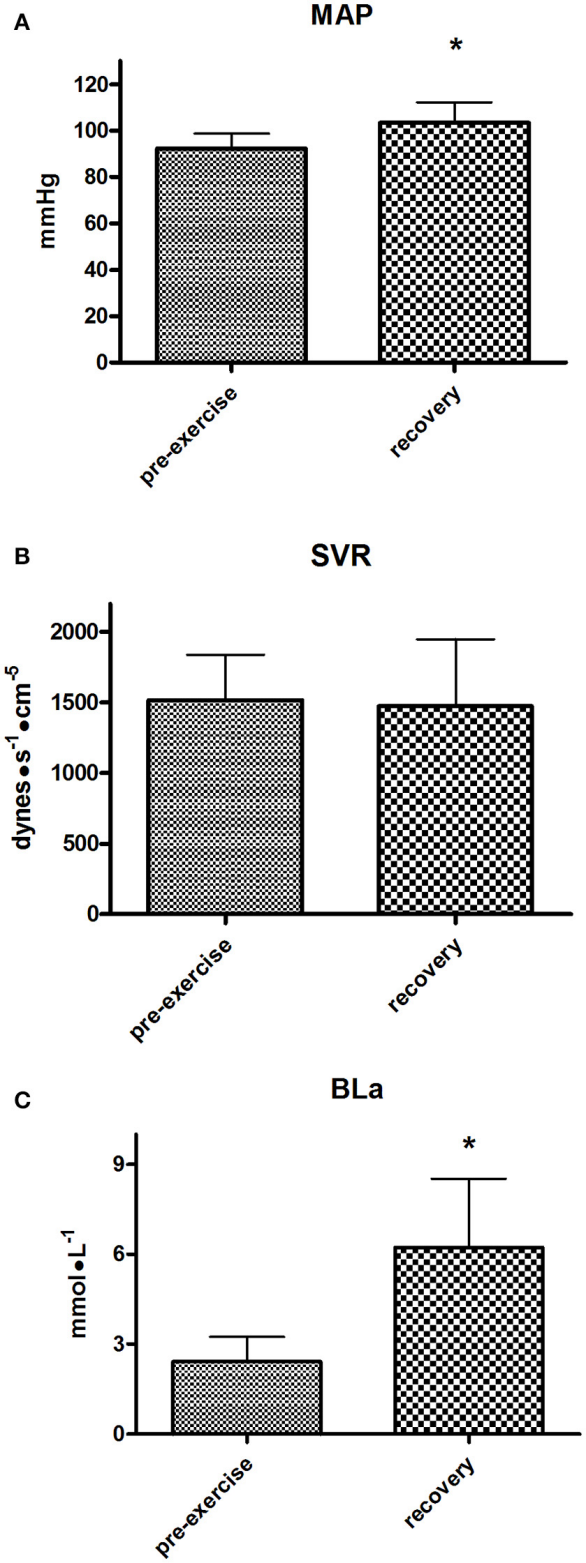
Group mean arterial pressure (MAP, **A**), systemic vascular resistance (SVR, **B**), and blood lactate (BLa, **C**) during the pre-exercise and recovery from the Enduro session. Values are mean ± SD (*n* = 15). ^*^p < 0.05 vs. pre-exercise.

Figures 3–**5** show the statistics results applied to each variable. HR significantly increased from 96.5 ± 12.8 bpm at rest to 153.1 ± 17.7 bpm during the experimental trial. This parameter reached peaks of up to 200 bpm in some subjects. Recovery led to a reduction in HR but it did not return to pre-exercise levels (Figure 3A). SV increased from a level of 53.5 ± 14.1 ml at rest to 72.2 ± 22.1 ml during the Enduro session, showing peaks of up to 125 ml. During recovery SV returned to pre-exercise levels (Figure 3B). Likewise, CO increased during exercise as compared to rest (5.0 ± 1.1 vs. 10.9 ± 3.0 L·min^−1^) to return to baseline during recovery (Figure 3C). In some subjects, CO reached a level of 20 L·min^−1^ during riding.

VER was enhanced during the riding session and it returned to pre-exercise levels during recovery. The average VER levels during rest and riding were 192.9 ± 43.0 and 324.1 ± 83.6 ml·s^1^ respectively, with peaks of up to 500 ml·s^−1^ during the experimental trial (Figure 4A). VFR was augmented by Enduro riding with respect to rest (849.1 ± 309.8 vs. 141.1 ± 160.5 ml·s^−1^). Peaks in VFR reached values of up to 1,500 ml·s^−1^. During recovery, this variable returned to values similar to those at pre-exercise (Figure 4B). Figure 4C demonstrates that PEP decreased during riding with respect to rest and that this decrement was still present during recovery. VET (Figure 4D) decreased during riding, but it returned to values close to pre-exercise during recovery. Moreover DT (Figure 4E) was greatly reduced by ridding to return to a level similar to pre-exercise during recovery.

MAP was higher during recovery than during pre-exercise, while SVR did not show any difference between pre-exercise and recovery (Figures 5A,B respectively). Finally, BLa significantly accumulated during recovery with respect to pre-exercise levels, reaching values higher than 6 mmol•L^−1^, with peaks of about 10 mmol•L^−1^ (Figure 5C).

## Discussion

The hemodynamic scenario obtained from the present study reveals that during Enduro a substantial increase in CO occurs. On average, this parameter doubled during riding from about 5 to 10 L·min^−1^ as compared to rest. This CO response was the consequence of a significant increase in both HR and SV. It should to be underscored that athletes performed not a real race. Instead, it was a short training, without the psychological stress imposed by a real race. Moreover, the riders performed the test alone, without rivals. Thus, any bias due to psychological stress can be ruled out.

Whilst an increment in HR is a well-known phenomenon during racing, and was expected, the increment in SV is an important new finding. To the best of our knowledge, this is the first time this parameter has been investigated during motocross riding. In detail, SV increased from an average pre-exercise level of 53 to 72 ml during riding, i.e., an increment of about 35%. This increment occurred notwithstanding the fact that motocross is primarily considered an activity where isometric actions prevail over dynamic actions (Konttinen et al., 2007). It is well-known that static exercise causes a reduction in SV, although conflicting results have been reported in the literature (Shoemaker et al., 2007; Elstad et al., 2009; Toska, 2010). Our hypothesis was that isometric strains impaired venous return, thereby reducing cardiac pre-load and SV. Furthermore, isometric strains caused an increase in after-load, which opposed ventricular emptying. Our reasoning was that if static effort prevailed over dynamic effort in Enduro, a reduction of, or a stable SV value throughout the race would have been expected. However, this was not observed in this study.

Several factors may explain this phenomenon. Firstly, it is possible that dynamic muscle contractions were as frequent as the isometric contractions during Enduro. Dynamic muscle activity facilitates venous return by squeezing peripheral veins, especially in the lower limbs (Carter et al., 1999; Crisafulli et al., 2004). Hence, we speculate that in our setting, the presence of dynamic muscle contractions counteracted the isometric activity, thereby improving cardiac filling and pre-load. This fact recruited the Frank-Starling mechanism and supported the SV response. Secondly, it must be remembered that venous return and cardiac pre-load also depend on the sympathetic activity. Indeed, during exercise, sympathetic-mediated venous-constriction takes place in order to centralize blood volume and to support SV when the mechanical and the metabolic arm of the exercise pressor reflex is activated, i.e., during eliciting of the so-called mechano-metaboreflex (Crisafulli et al., 2009; Nóbrega et al., 2014; Milia et al., 2015). This reflex, provides continuous feedback on the mechanical and metabolic status of contracting muscles and is stimulated by both isometric and dynamic exercise. Hence, it is possible to hypothesize that muscle activity occurring during Enduro activated the mechano-metaboreflex, thereby leading to a sympathetic-mediated venous constriction, which in turn, enhanced venous return and cardiac pre-load. This resulted in the augmented SV observed in the present investigation. This occurrence is supported by the findings that VFR was substantially increased during the riding period. This parameter is the measure of the filling rate of ventricles and is an expression of the global capacity of the circulatory system to support pre-load during diastole (Gledhill et al., 1994; Crisafulli et al., 2000; Marongiu et al., 2013; Milia et al., 2014). It is to be noticed that this mechanism was very effective since diastole shortened more than ejection time, thereby reducing the time available for ventricular filling. Actually, the increase in VFR was greater than that in VER.

Another phenomenon that could explain the SV response was the enhanced cardiac performance, as testified by VER behavior. This parameter is the rate of ventricle emptying is correlated to cardiac performance (Tanaka et al., 1986; Concu and Marcello, 1993; Gledhill et al., 1994). Our finding of an enhancement of VER during riding demonstrates that an increase in myocardial performance takes place during Enduro and this fact, along with the increased VFR, further supports the rise in SV found in the present investigation. Two phenomena can account for the improved myocardial performance: (i) the recruitment of the Frank-Starling mechanism by the augmented cardiac pre-load due to the sustained VFR; and (ii) the sympathetic-mediated increase in inotropism.

The hypothesis that the sympathetic tone was elevated during Enduro is supported by HR time course, which showed a mean group level of 153 bpm, with peaks of up to 200 bpm in some individuals. The sympathetic activation was likely the consequence of the recruitment of both the central command and the exercise pressor reflex, which are operative during exercise even at mild levels (Nóbrega et al., 2014; Crisafulli et al., 2015).

Taken together, data from the present study support the concept that Enduro is a challenging activity from a cardiovascular point of view. Actually, substantial hemodynamic activation occurred during the 10 min of riding utilized in the present study. Thus, we can speculate that this hemodynamic engagement would have been even higher if riding sessions had been longer. Moreover, it also emerged that the cardiovascular apparatus was intermittently activated. Figure 2 shows that SV and CO peaked several times throughout the race, whereas this phenomenon was not evident looking at the HR time course alone. Another outcome that deserves attention is that substantial metabolic engagement was also present, as is supported by the BLa level gathered immediately after the races.

### Limitations of the study

In the present investigation the impedance cardiography method was used to collect hemodynamics. This method has been found reliable in healthy subjects during exercise and recovery, although some concerns still remain on its reliability (Warburton et al., 1999; Charloux et al., 2000; Richard et al., 2001; Crisafulli et al., 2003a). The main concern with this technique is that leg and chest movements may render reference points on impedance traces unrecognizable, thereby affecting the reliability of the method. In order to overcome this problem in the present investigation, impedance signals were digitally reordered and then analyzed offline by a skilled operator who inspected the stored signals and rejected traces affected by artifacts. Hence, hemodynamic variables were derived from readable impedance waveforms only. This signal processing procedure has been used several times in our laboratory in various kinds of exercise activities (Crisafulli et al., 2003a, 2004, 2006b, 2008) and, although time-consuming, it allowed us to obtain reliable and reproducible hemodynamic data estimations in different experimental settings, including extreme environments such as diving in the sea (Tocco et al., 2013; Marongiu et al., 2015). Moreover, the aim of this work was not to study absolute values, rather, to evaluate relative changes in hemodynamics since we were not interested in comparing two groups of subjects. Even though the impedance method is not quantitatively accurate, it has been demonstrated to provide reliable qualitative estimations of cardiovascular changes during exercise and recovery (Tanaka et al., 1986; Concu and Marcello, 1993; Charloux et al., 2000; Crisafulli et al., 2000; Richard et al., 2001).

Another potential limitation is that we did not measure sympathetic activity. The direct measure of sympathetic tone was of course not an option in our setting. Thus, the only possibility was to indirectly assess sympathetic tone. One option could be the analysis of HR power spectral analysis. However, this method requires stable HR conditions, i.e., without abrupt changes in HR, which was not the case in our setting. A further option could be the dosage of catecholamine spillover, however, this is not possible in our laboratory.

A further potential limitation of the present study was that, although participants underwent an incremental exercise test, we did not collect data related to athletes' aerobic fitness, such as VO_2max_. Aerobic fitness is potentially a precursor to Enduro performance, especially for long-lasting competitions. However, the object of this study was the cardiovascular stress imposed by acute motorcycle riding and not endurance capacity. Moreover, the maximum workload level reached by athletes during the preliminary exercise test indicated a medium level of maximum aerobic capacity, which was comparable to what had been previously reported for soccer players performing the same test in our laboratory (Crisafulli et al., 2004).

A final potential limitation were that heat production and hydration status of subjects were not determined. As previously stated, the 10-min period of motorcycle trial was chosen to avoid cardiovascular perturbations also taking into consideration the potential occurrence of dehydration and thermal stress. It was in fact unlikely that this short motorcycle session could cause cardiovascular impairment ought to fluids loss and excessive body temperature. It was reported that short aerobic exercise (15 min cycling) in two environmental conditions (20 and 40°C) yielded similar rating of perceived exertion, HR, and core body temperature (Ely et al., 2009). Moreover, competitive runners are often observed to achieve body temperature >40°C without apparent sequelae (Byrne et al., 2006). Furthermore, significant cardiovascular effects of dehydration occur when water deficit is >3% of total body water. It has been observed that if a subject starts activity in a normal state of hydration, then dehydration-mediated performance decrements are restricted to activities lasting 1 h or longer, since sustainable exercise sweating are typically <1.5 l/h (Cheuvront et al., 2010). Hence, in our opinion the brief motorcycle sessions of the present study could not cause a dehydration able to impair the cardiovascular functions.

#### Perspective

It should be considered that the 10-min period of motorcycle trial used in the present research did not allow us to draw conclusions on hemodynamic consequences of longer Enduro sessions. In these situations, cardiovascular stress is supposed to be even higher because of the presence of thermal stress and dehydration. It is well known that the combination of exercise, heat stress, and dehydration can impose severe challenges on the cardiovascular apparatus. The increased skin blood flow necessary for heat dispersion together with fluid loss cause a reduction in cardiac pre-load, thereby reducing SV and eliciting cardiac drift (Crisafulli et al., 2006a). This phenomenon is particularly evident when the total body water loss is >3% (Cheuvront et al., 2010; González-Alonso, 2012). Our protocol was devised to study acute hemodynamic changes rather than the effects of long-lasting races. Indeed, it was unlikely that the short protocol employed (10 min) could have caused significant reductions in cardiac pre load and cardiac drift. Therefore, further research is needed to better depict the whole hemodynamic scenario during a typical long-lasting Enduro race.

In conclusion, the hemodynamic scenario found in the present investigation supports the hypothesis that Enduro racing leads to the activation of the central command and the exercise pressor reflex, which, together with HR increments, recruited cardiac performance, pre-load, and after-load. These facts together concurred in increasing stroke volume and cardiac output during racing. The described hemodynamic activation may represent a hemodynamic challenge as, in some individuals, HR reached values of up to 200 bpm and CO achieved levels of about 20 L·min^−1^. Finally, substantial metabolic engagement was present as well, as BLa showed peaks of 10 mmol•L^−1^.

## Author contributions

IS, VP, RM, and AC conceived the study, conducted experiments, designed and wrote the paper. GM, SR, and SO conducted experiments, designed and wrote the paper. All authors read and approved the final version of the manuscript.

### Conflict of interest statement

The authors declare that the research was conducted in the absence of any commercial or financial relationships that could be construed as a potential conflict of interest.
